# Patient-Specific Human Induced Pluripotent Stem Cell Model Assessed with Electrical Pacing Validates S107 as a Potential Therapeutic Agent for Catecholaminergic Polymorphic Ventricular Tachycardia

**DOI:** 10.1371/journal.pone.0164795

**Published:** 2016-10-20

**Authors:** Kenichi Sasaki, Takeru Makiyama, Yoshinori Yoshida, Yimin Wuriyanghai, Tsukasa Kamakura, Suguru Nishiuchi, Mamoru Hayano, Takeshi Harita, Yuta Yamamoto, Hirohiko Kohjitani, Sayako Hirose, Jiarong Chen, Mihoko Kawamura, Seiko Ohno, Hideki Itoh, Ayako Takeuchi, Satoshi Matsuoka, Masaru Miura, Naokata Sumitomo, Minoru Horie, Shinya Yamanaka, Takeshi Kimura

**Affiliations:** 1 Department of Cardiovascular Medicine, Kyoto University Graduate School of Medicine, Kyoto, Japan; 2 Kyoto University iPS Cell Research and Application, Kyoto, Japan; 3 Department of Cardiovascular and Respiratory Medicine, Shiga University of Medical Science, Otsu, Japan; 4 Department of Integrative and Systems Physiology, Faculty of Medical Sciences, University of Fukui, Fukui, Japan; 5 Division of Cardiology, Tokyo Metropolitan Children’s Medical Center, Tokyo, Japan; 6 Department of Pediatric Cardiology, Saitama Medical University International Medical Center, Saitama, Japan; Indiana University, UNITED STATES

## Abstract

**Introduction:**

Human induced pluripotent stem cells (hiPSCs) offer a unique opportunity for disease modeling. However, it is not invariably successful to recapitulate the disease phenotype because of the immaturity of hiPSC-derived cardiomyocytes (hiPSC-CMs). The purpose of this study was to establish and analyze iPSC-based model of catecholaminergic polymorphic ventricular tachycardia (CPVT), which is characterized by adrenergically mediated lethal arrhythmias, more precisely using electrical pacing that could promote the development of new pharmacotherapies.

**Method and Results:**

We generated hiPSCs from a 37-year-old CPVT patient and differentiated them into cardiomyocytes. Under spontaneous beating conditions, no significant difference was found in the timing irregularity of spontaneous Ca^2+^ transients between control- and CPVT-hiPSC-CMs. Using Ca^2+^ imaging at 1 Hz electrical field stimulation, isoproterenol induced an abnormal diastolic Ca^2+^ increase more frequently in CPVT- than in control-hiPSC-CMs (control 12% vs. CPVT 43%, p<0.05). Action potential recordings of spontaneous beating hiPSC-CMs revealed no significant difference in the frequency of delayed afterdepolarizations (DADs) between control and CPVT cells. After isoproterenol application with pacing at 1 Hz, 87.5% of CPVT-hiPSC-CMs developed DADs, compared to 30% of control-hiPSC-CMs (p<0.05). Pre-incubation with 10 μM S107, which stabilizes the closed state of the ryanodine receptor 2, significantly decreased the percentage of CPVT-hiPSC-CMs presenting DADs to 25% (p<0.05).

**Conclusions:**

We recapitulated the electrophysiological features of CPVT-derived hiPSC-CMs using electrical pacing. The development of DADs in the presence of isoproterenol was significantly suppressed by S107. Our model provides a promising platform to study disease mechanisms and screen drugs.

## Introduction

Catecholaminergic polymorphic ventricular tachycardia (CPVT) is a hereditary arrhythmic disorder characterized by bidirectional ventricular tachycardia (VT) that is triggered by emotional stress or physical exercise and leads to syncope or sudden cardiac death without structural heart disease. CPVT is caused by autosomal dominant mutations in the cardiac ryanodine receptor gene (*RyR2*) [1], the calmodulin gene (*CALM1*, *CALM2*) [2, 3] and the inward rectifying potassium channel gene (*KCNJ2*) [4] and autosomal recessive mutations in the cardiac calsequestrin gene (*CASQ2*) [5] and the triadin gene (*TRDN*) [6]. Approximately 50–55% of CPVT cases are associated with *RyR2* mutations [7] and 1–2% are due to *CASQ2* mutations [8]. Beta-blockers are the first-line therapy for CPVT, but they often fail to prevent fatal arrhythmias [9]. Recently, flecainide, a class Ic Na^+^ channel blocker, has been reported to be effective for treating CPVT patients [10].

The advent of human induced pluripotent stem cell (hiPSC) technology has enabled us to use human cardiomyocytes that have the same genetic background as the patients. There have been several reports of CPVT iPSC-based model [11–15], however, it is becoming clearer that hiPSC-derived cardiomyocytes (hiPSC-CMs) have immature electrophysiological and structural properties compared to adult human cardiomyocytes [16, 17], which hamper us to analyze their phenotype precisely. In the present study, we demonstrated that hiPSC-CMs beat in an irregular disorganized pattern even in those of control, and suggested the usefulness of electrical pacing during Ca^2+^ transient and action potential (AP) recordings in hiPSC-CMs. In addition, we investigated the efficacy of S107, a 1,4-benzothiazepine derivative that is a promising candidate drug for treating CPVT. S107 was discovered by Marks et al and reported to correct leaky RyR1, 2 by stabilizing interactions between RyR 1, 2 and calstabin 1, 2 [18]. The potency of S107 for preventing the development of DADs in CPVT-hiPSC-CMs indicates that our model could be useful for investigating new pharmacotherapies.

## Materials and Methods

### hiPSC generation and cardiomyocyte differentiation

Human dermal fibroblasts were obtained from a patient after written informed consent was obtained. The fibroblasts were retrovirally transduced with a combination of 4 transcription factors (Oct3/4, Sox2, Klf-4, c-Myc) to generate hiPSCs. This study was approved by Kyoto University ethics review board (G259) and conformed to the Declaration of Helsinki. All patients provided written informed consent. The control hiPSC line, 201B7, was generated from a healthy individual using the same transcription factors [19]. The hiPSCs were differentiated into cardiomyocytes using an embryoid body (EB) differentiating system described previously [20]. For Ca^2+^ imaging, EBs were treated with collagenase B (Roche, Indianapolis, IN, USA) and trypsin EDTA (Nacalai Tesque, Kyoto, Japan) at day 21 of differentiation and dispersed into single cells or small clusters which were plated onto gelatin-coated dishes. For gene expression analyses, EBs were plated onto fibronectin-coated dishes without dissociation at day 21. After being plated on dishes, hiPSC-CMs were maintained in culture medium consisting of DMEM/F12 supplemented with 2% fetal bovine serum, 2 mmol/L L-glutamine, 0.1 mmol/L non-essential amino acids, 0.1 mmol/L β-mercaptoethanol, 50 U/ml penicillin, and 50 μg/ml streptomycin [21]. The medium was renewed every 2–3 days.

### Genomic sequencing and karyotyping

Genomic DNA was isolated from control and CPVT-hiPSC lines by GenElute Mammalian Genomic DNA Miniprep kit (Sigma-Aldrich, St Louis, MO, USA). Purified DNA was amplified with specific primers and analyzed by 3100 Genetic Analyzer and Big Dye Terminator v1.1 (Applied Biosystems, Foster City, CA, USA). Chromosomal G-banding analysis was performed using a standard procedure (Nihon Gene Research Laboratories, Sendai, Japan). Primers are detailed in S1 Table.

### Immunocytochemistry

The hiPSC colonies were fixed in 4% paraformaldehyde (PFA) for 20 min. The cells were permeabilized in 0.2% Triton X-100 (Nacalai Tesque). The samples were stained with the following primary antibodies: mouse monoclonal anti-OCT3/4 (1:50; Santa Cruz Biotechnology, Delaware, CA, USA), mouse monoclonal anti-SSEA4 (1:200; Santa Cruz Biotechnology), and mouse monoclonal anti-TRA 1–60 (1:200; Santa Cruz Biotechnology). The secondary antibody was donkey anti-mouse Alexafluor 488 (1:1000, Invitrogen, Carlsbad, CA, USA). The nuclei were stained with DAPI (1:2000, Wako Pure Chemical Industries, Osaka, Japan). The specimens were observed under a fluorescence microscope (Biozero BZ-9000; KEYENCE, Osaka, Japan).

### Teratoma formation

In order to determine the pluripotency of hiPSCs, we performed teratoma formation in immune-compromised mice. Two NOD/SCID male mice were maintained under a 12 hours light / 12 hours dark cycle and fed *ad libitium*. Mice were inspected daily by the veterinary staff. Following anesthesia with pentobarbital (50 mg/kg), the hiPSCs were injected as cell clumps into NOD/SCID mice under the testis capsule. The injection site was monitored for tumor growth weekly. Tumor samples were collected at 8 weeks, fixed in 10% formalin and stained with hematoxylin and eosin. Mice were euthanized by cervical dislocation. All animal experiments were performed in accordance with the ‘Guide for the Care and Use of Laboratory Animals’ (2011) of the National Institutes of Health and the Regulation on Animal Experimentation at Kyoto University, and approved by Ethics Committee of Kyoto University (Permit Number: kei 31–18).

### Analysis of mRNA expression by real-time quantitative polymerase chain reaction (qPCR)

Total RNA was isolated using TRIzol Reagent (Invitrogen, Carlsbad, CA, USA) from 20 to 30 spontaneously beating EBs microdissected at day 30 and day 90, and treated with TURBO DNA-free Kit (Applied Biosystems, Foster City, CA, USA). Total RNA from human whole heart tissue (BioChain Institute, Newark, CA, USA) was also reverse transcribed into complementary DNA (cDNA) for comparison. The cDNA was synthesized from 1 μg of total RNA, in a total volume of 20 μl, using oligo (dT)_18_ primer with Transcriptor First Strand cDNA Synthesis Kit (Roche). TaqMan real-time PCR assay was performed using FastStart Universal Probe Master (Rox) and an appropriate probe from Universal ProbeLibrary Set (Roche). The expression of genes of interest was normalized to that of *GAPDH*. Relative quantification was calculated according to the ΔΔC_T_ method. The changes in gene expression levels were compared with those of adult human heart. The fold change is expressed as mean ± SEM. A list of the primers used in these experiments is provided in S1 Table.

### Ca^2+^ imaging

The hiPSC-CMs were dispersed with collagenase B and Trypsin EDTA and plated onto glass coverslips coated with fibronectin (BD Biosciences, San Jose, CA, USA). After 5–7 days, dissociated hiPSC-CMs on a coverslip were loaded with 2 μmol/L Fluo-8 (AAT Bioquest, Sunnyvale, CA, USA) in the culture medium described above. After incubation for 30 min at 37°C in 5% CO_2_, the medium was replaced with normal tyrode solution containing (in mmol/L): 140 NaCl, 0.33 NaH_2_PO_4_, 5.4 KCl, 1.8 CaCl_2_, 0.5 MgCl_2_, 5.0 HEPES, and 5.5 D-Glucose. Spontaneously contracting or electrically stimulated single cells were analyzed at 36.0 ± 1.0°C. The imaging of fluo-8 was analyzed for average pixel intensities of regions of interest drawn to include whole cell, following background correction, using an Aquacosmos image-processing system (Hamamatsu Photonics, Hamamatsu, Japan). The hiPSC-CMs were stimulated at 0.5 and 1.0 Hz with 4 ms depolarizing pulses at 20–30 V using platinum electrodes, with an interelectrode distance of 12 mm (Intermedical, Osaka, Japan). The rhythmicity of the spontaneous Ca^2+^ transient was assessed by calculating the cycle length variability index, defined as the standard deviation of the cycle length/mean cycle length. A solution containing 100 nM isoproterenol (LKT Laboratories, Saint Paul, MN, USA) was prepared fresh before the experiment and applied 5–10 min before data collection. A 10 μM ryanodine solution (Wako Pure Chemical Industries) was added 2–3 min before isoproterenol administration.

### Electrophysiological recordings

The hiPSC-CMs were enzymatically dissociated with collagenase B and Trypsin EDTA and plated onto glass coverslips coated with fibronectin (BD Biosciences). APs were recorded at 36.0 ± 1.0°C in a current clamp mode using a perforated patch-clamp technique. The pipette solution contained 300 μg/ml amphotericin B (Sigma-Aldrich, St Louis, MO, USA) and the following (in mM): 150 KCl, 5 EGTA, 5 MgATP, 10 HEPES, 5 NaCl, 2 CaCl_2_, pH was adjusted to 7.2 by KOH. The experiments were performed under continuous perfusion of the extracellular solution containing (in mM): 150 NaCl, 5.4 KCl, 1.8 CaCl_2_, 1 MgCl_2_, 15 glucose, 15 HEPES, 1 Na-pyruvate (pH adjusted to 7.40 with NaOH) [22]. Patch-clamp pipettes, formed from borosilicate glass with PP-830 (Narishige, Tokyo, Japan) and had a resistance of 4–7 MΩ. APs were recorded from spontaneously contracting and quiescent hiPSC-CMs. All signals were acquired at 10 kHz, digitized with a Digidata 1332A (Axon instruments, CA, USA) and analyzed with a pCLAMP 10.4 software (Axon instruments). Current clamp recordings were performed using a MultiClamp 700B amplifier. Solutions containing 100 nM isoproterenol were applied using a gravitational flow system 5–10 min prior to data collection. We added 1 and 10 μM freshly prepared S107 (Cayman Chemical, Ann Arbor, MI, USA) to the culture medium 2–3 hours prior to the experiments.

### Statistical analysis

Continuous variables are presented as the mean ± SEM. Categorical variables are expressed as frequencies. Differences in the means between two groups were compared using Student’s t-tests. Categorical differences between two groups were evaluated using chi-squared tests. A value of p < 0.05 was considered statistically significant.

## Results

### Generation of patient-specific CPVT-hiPSCs

We generated hiPSCs from a 37-year-old female patient with CPVT, whose clinical features were previously reported [23]. She first experienced syncope during running or swimming at 15 years of age. Her next episodes of syncope were while riding a bicycle and when under emotional stress caused by a traffic accident at 30 years of age. She then remained free of syncope until the age of 36 years when she experienced an episode upon awakening from a nightmare, at which time she was admitted to the hospital. Polymorphic VT was recorded during an exercise test and an epinephrine provocation test. She was diagnosed with CPVT and began taking carvedilol. However, she had an episode of syncope and polymorphic VT was recorded again during an exercise test despite having received an oral beta-blocker. Subsequently, she underwent implantation of an implantable cardioverter-defibrillator (ICD). At the age of 37, an appropriate ICD discharge occurred and she began taking flecainide, which was effective in preventing polymorphic VT. Genetic analyses identified a missense mutation, c.13759 A>G, p.I4587V, in the *RyR2* gene. Her son also had a history of syncope and was a carrier of an identical mutation.

**Fig 1:**
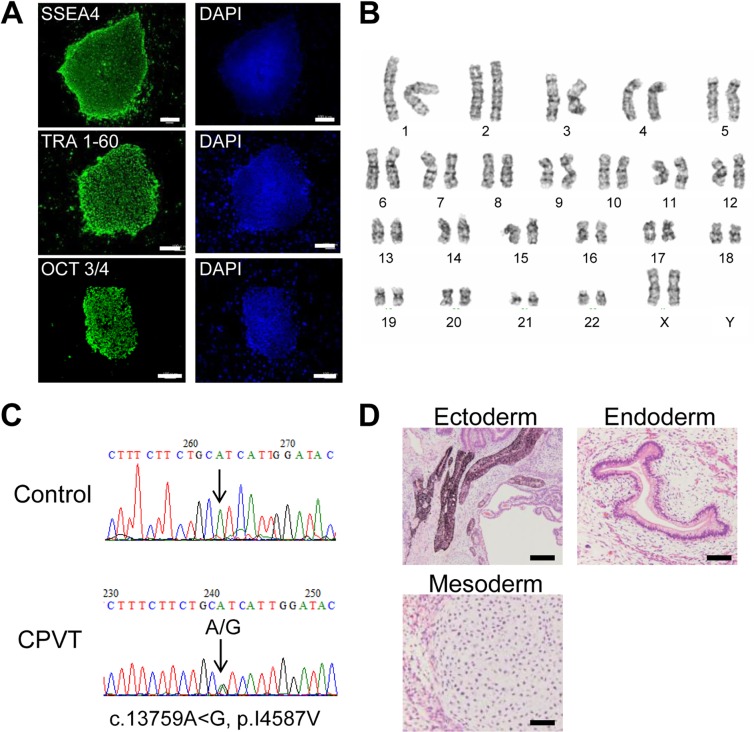
Characterization of CPVT-hiPSCs. (A) CPVT-hiPSC colonies derived from the dermal fibroblasts of a patient with CPVT expressed pluripotency markers, shown by immunostaining. Scale bars = 200 μm. (B) CPVT-hiPSCs maintained the normal karyotype. (C) Sequencing analysis of the *RYR2* gene identified the I4587V heterozygous point mutation in the CPVT-hiPSCs. (D) Hematoxylin-eosin staining of teratomas formed from CPVT-hiPSCs showed differentiation of the cells into various tissues derived from all three germ layers: pigmented epithelium (ectoderm), gut-like structures (endoderm), and cartilage tissue (mesoderm). Scale bar = 100 μm.

Dermal fibroblasts were obtained from the proband and retrovirally transduced with 4 genes (Oct3/4, Sox2, Klf-4, and c-Myc). As a control, we used an hiPSC line, 201B7, which was similarly generated from a healthy individual [19]. The CPVT-hiPSCs exhibited characteristic human embryonic stem cell morphology and stained positively for pluripotency markers (OCT3/4, SSEA4, TRA1-60) (Fig 1A). The CPVT-hiPSC lines displayed a normal karyotype (Fig 1B). We confirmed the *RyR2*-I4587V mutation in the CPVT-hiPSCs, but not in the control-hiPSCs (Fig 1C). In order to confirm the pluripotency of generated hiPSCs, we injected hiPSCs into severe combined immunodeficiency (SCID) mice, which led to the formation of teratomas, containing tissue derivatives of three germ layers: pigmented epithelium (ectoderm), gut-like structures (endoderm), and cartilage tissue (mesoderm) (Fig 1D).

### Gene expression of hiPSC-CMs

**Fig 2:**
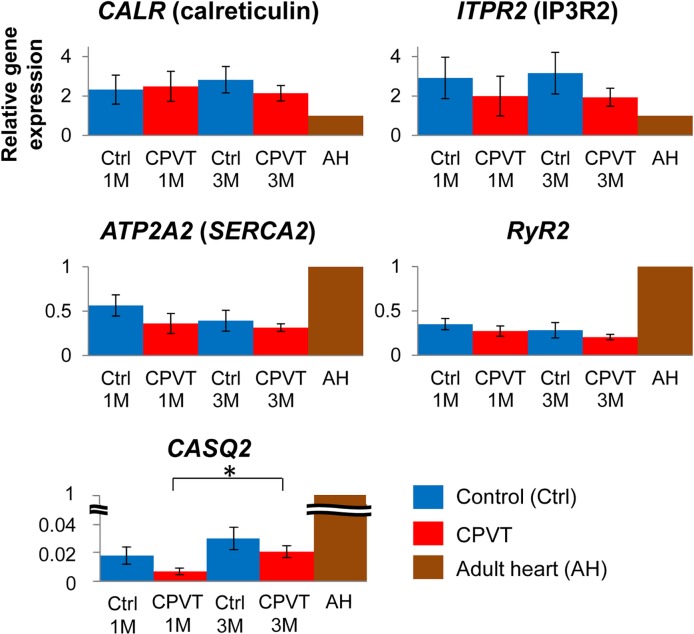
Gene expression of calcium handling proteins in hiPSC-CMs. Quantitative real-time PCR of spontaneously contracting embryoid bodies differentiated from control- and CPVT-hiPSCs showed comparable expression levels of the studied calcium handling proteins. All values are relative to the adult human heart and were normalized to glyceraldehyde 3-phosphate dehydrogenase (GAPDH). PCR = polymerase chain reaction

We differentiated control- and CPVT-hiPSCs into spontaneously contracting cardiomyocytes using an EB differentiation system. Spontaneously beating EBs started to appear at day 7 of differentiation. EBs were plated on fibronectin-coated dishes at day 21 and maintained in the culture medium described above for an additional 2 months. We conducted quantitative real-time PCR in control- and CPVT-hiPSC-CMs at days 30 and 90, and the expression levels of the genes involved in Ca^2+^ handling are shown in Fig 2. Consistent with previously reported transcriptional profile data on human iPSC/ESC-derived cardiomyocytes [24], *CASQ2* expression levels were extremely low compared to those of adult human cardiomyocytes. However, *RyR2* and *SERCA2* were already expressed at 1 month similar to adult human myocytes in the control- and CPVT-hiPSC-CMs. *Innositol-1*,*4*,*5-trisphosphate receptor 2* (*IP3R2)* and *calreticulin* were highly expressed in the control- and CPVT-hiPSC-CMs compared to adult human cardiomyocyts (Fig 2). In this study, there was no significant difference in Ca^2+^ handling gene expression between control- and CPVT-hiPSC-CMs.

### CPVT-hiPSC-CMs presented diastolic Ca^2+^ waves with adrenergic stimulation upon Ca^2+^ imaging

**Fig 3:**
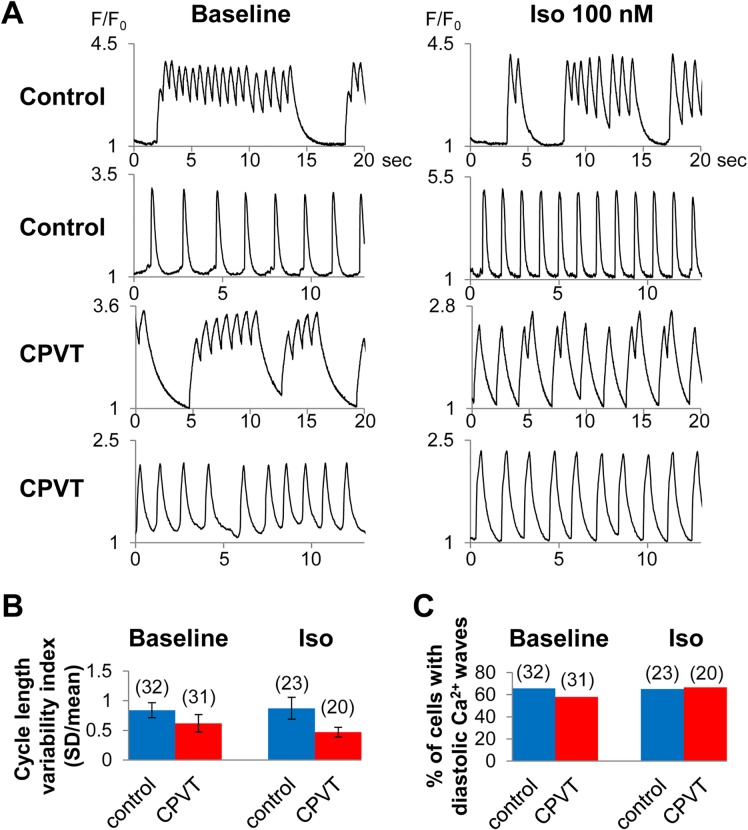
Ca^2+^ imaging of spontaneously beating hiPSC-CMs. (A) Representative tracings of Ca^2+^ imaging in spontaneously contracting control- and CPVT-hiPSC-CMs. (B) Cycle length variability indices, defined as the standard deviation of the cycle length/mean cycle length of spontaneously beating hiPSC-CMs showed no significant difference between control and CPVT. (C) The percentage of spontaneously contracting control- and CPVT-hiPSC-CMs presenting diastolic Ca^2+^ waves at baseline and after isoproterenol administration.

We performed Ca^2+^ imaging of spontaneously contracting control- and CPVT-hiPSC-CMs. A considerable number of both control- and CPVT-hiPSC-CMs showed irregular beating rhythms at baseline and after isoproterenol administration (Fig 3A). To evaluate the irregularity parameter, we calculated the cycle length variability index, also known as the coefficient of variation, defined as the cycle length standard deviation divided by the mean of the cycle length. The cycle length variability indices were higher in hiPSC-CMs compared to those in isolated rabbit nodal cells [25], but there was no significant difference between control and CPVT cells (Fig 3B). In spontaneous beating hiPSC-CMs, we found no significant difference in the frequency of diastolic Ca^2+^ waves (increased intracellular Ca^2+^ concentration during the diastolic phase) between control and CPVT cells (Fig 3C).

**Fig 4:**
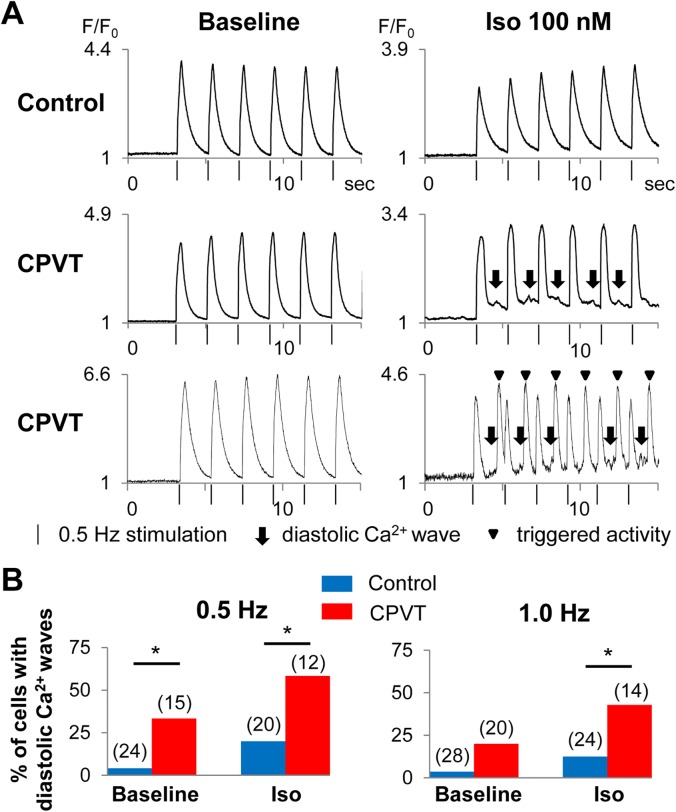
Ca^2+^ imaging of electrically stimulated hiPSC-CMs. (A) Representative tracings of Ca^2+^ imaging in control- and CPVT-hiPSC-CMs with electrical stimulation at 0.5 Hz. Arrows indicate the diastolic Ca^2+^ waves and arrowheads indicate the triggered activity. Vertical bars indicate the time points that the hiPSC-CMs were stimulated at 0.5 Hz. (B) The percentage of control- and CPVT-hiPSC-CMs presenting diastolic Ca^2+^ waves at baseline and after isoproterenol administration (at 0.5 and 1 Hz pacing). *p < 0.05.

Therefore, to minimize the effect of beating intervals, we used electrical field stimulation when recording Ca^2+^ transients to assess the arrhythmic phenotype in hiPSC-CMs (Fig 4A). At baseline, CPVT-hiPSC-CMs showed diastolic Ca^2+^ waves significantly more frequently than control-hiPSC-CMs at 0.5 Hz pacing (CPVT: 33%, control: 4%; p < 0.05; Fig 4B). After administration of 100 nM isoproterenol, significantly more frequent diastolic Ca^2+^ waves were recorded in CPVT-hiPSC-CMs than control-hiPSC-CMs (CPVT: 58%, control: 20% at 0.5 Hz; p < 0.05; CPVT: 43%, control: 12% at 1 Hz; p < 0.05; Fig 4B).

**Fig 5:**
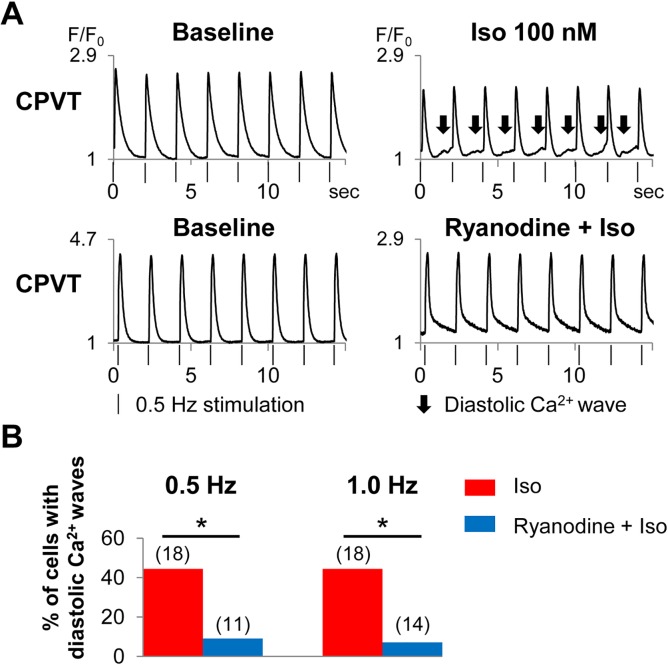
Ryanodine suppressed diastolic Ca^2+^ waves in CPVT-hiPSC-CMs. (A) Representative tracings of Ca^2+^ imaging in CPVT-hiPSC-CMs with (lower) and without (upper) ryanodine. Note diastolic Ca^2+^ waves (arrows) without ryanodine (upper, right), however, no diastolic Ca^2+^ waves with ryanodine (lower, right). Vertical bars indicate the time points that the CPVT-hiPSC-CMs were stimulated at 0.5 Hz. (B) Fraction (in %) of CPVT-hiPSC-CMs that showed diastolic Ca^2+^ waves with and without ryanodine. *p < 0.05.

We treated the CPVT-hiPSC-CMs with ryanodine, a strong ligand of the ryanodine receptor (Fig 5A). With 10 μM ryanodine, the percentage of CPVT-hiPSC-CMs that presented diastolic Ca^2+^ waves after isoproterenol administration significantly decreased to 9% at 0.5 Hz and 7% at 1 Hz pacing (Fig 5B).

### β-adrenergic stimulation-induced DADs in CPVT-hiPSC-CMs

**Fig 6:**
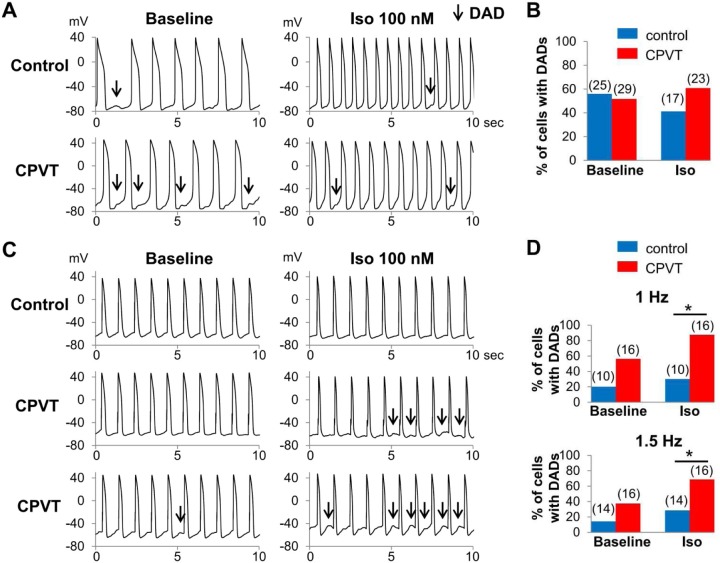
AP recordings of hiPSC-CMs. (A) Representative tracings of AP recordings from spontaneously beating control- and CPVT-hiPSC-CMs. Both control- and CPVT-hiPSC-CMs showed DADs. (B) No significant difference was found in the percentage of spontaneously contracting hiPSC-CMs presenting DADs between control and CPVT. (C) Representative tracings of AP recordings during 1 Hz pacing from control- and CPVT-hiPSC-CMs. CPVT-hiPSC-CMs showed DADs, especially after isoproterenol administration. (D) The percentage of control- and CPVT-hiPSC-CMs that developed DADs at 1 and 1.5 Hz pacing. *p < 0.05.

The percentage of CPVT-hiPSC-CMs that developed DADs during AP recordings did not differ from that of control-hiPSC-CMs under the spontaneously beating condition at baseline and after isoproterenol administration (Fig 6A and 6B). Under regular electrical stimulation, AP recordings revealed no significant difference in the percentage of cardiomyocytes with DADs between control and CPVT at baseline (CPVT: 56%, control: 20% at 1 Hz; CPVT: 38%, control: 14% at 1.5 Hz; Fig 6C and 6D). However, administration of isoproterenol at 100 nM produced significantly more frequent DADs in CPVT-hiPSC-CMs than control-hiPSC-CMs (CPVT: 86%, control: 30% at 1 Hz; p < 0.05; CPVT: 69%, control: 29% at 1.5 Hz; p < 0.05; Fig 6D). We found no early afterdepolarization in the AP recordings of control- and CPVT-hiPSC-CMs when paced at 1 or 1.5 Hz.

### S107 exerted an antiarrhythmic effect on CPVT-hiPSC-CMs

**Fig 7:**
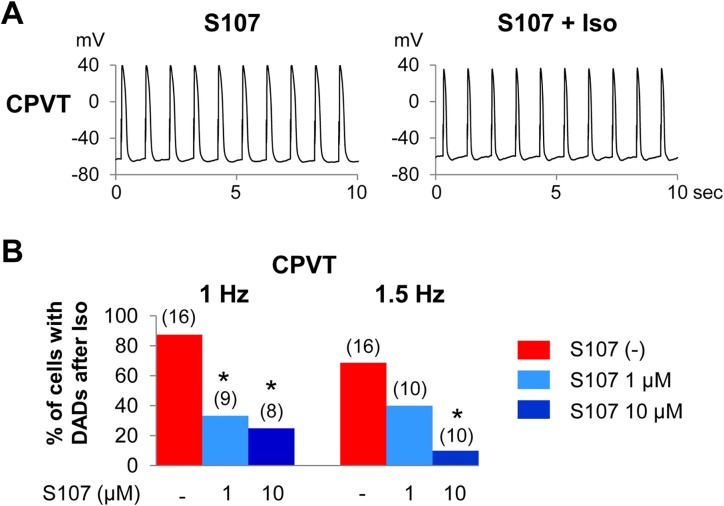
S107 prevented DADs in CPVT-hiPSC-CMs. (A) Representative tracings of AP recordings from CPVT-hiPSC-CMs following a 2–3 h pre-incubation with 10 μM S107. DADs were not found after isoproterenol application. (B) Fraction (in %) of CPVT-hiPSC-CMs that showed DADs after isoproterenol administration with and without S107 pre-incubation. S107 suppressed DADs in a concentration-dependent manner. *p < 0.05 versus the absence of S107.

We treated CPVT-hiPSC-CMs with the 1,4-benzothiazepine derivative S107. Pre-incubation with S107 for 2–3 hours suppressed the frequency of DADs in CPVT-hiPSC-CMs in a concentration-dependent manner (Fig 7A). Incubation with 1 μM S107 significantly decreased the percentage of CPVT-hiPSC-CMs presenting DADs to 33% at 1 Hz pacing after isoproterenol administration. Incubation with 10 μM S107 further decreased the percentage of CPVT-hiPSC-CMs with DADs compared to those lacking S107 (25% at 1 Hz; p < 0.05, 10% at 1.5 Hz; p < 0.05; Fig 7B).

## Discussion

Since the recognition of *RyR2* as a gene responsible for CPVT [1], functional analyses have suggested that DADs following Ca^2+^ leakage from the sarcoplasmic reticulum (SR) are associated with ventricular arrhythmia in CPVT; however, the mechanisms underlying SR Ca^2+^ leak have not been elucidated. Even now, some patients with CPVT experience syncope or sudden cardiac death. Although we must develop novel therapeutic approaches, it is uncertain whether the results of drug testing in mouse models are replicated in humans. Patient-specific hiPSC-based models of CPVT offer new opportunities for studying drug effects and pathogenetic mechanisms in human cardiomyocytes, however, it is not invariably successful to recapitulate the phenotypes due to the electrophysiological immaturity of hiPSC-CMs. In this study, we precisely evaluated the electrophysiological properties using electrical pacing, and confirmed abnormal diastolic Ca^2+^ waves during Ca^2+^ imaging and DADs during AP recordings in CPVT-hiPSC-CMs, which are two major phenotypes shown in CPVT knock-in mouse models [26, 27]. In addition, S107 prevented the development of DADs in CPVT-hiPSC-CMs.

Cardiomyocytes derived from human embryonic stem cells (hESC-CMs) have functional IP3-dependent Ca^2+^ release and the gene expression level of *IP3R2* progressively declines with the maturation of the hESC-CMs [28]. For the first time, we showed that *IP3R2* was highly expressed in hiPSC-CMs compared to adult human cardiomyocytes. IP3-dependent Ca^2+^ signaling has been shown to play an important role during the process of cardiac development. Calreticulin is also an important Ca^2+^ buffer and a regulator of Ca^2+^ homeostasis during fetal life. High expression levels of *IP3R2* and *calreticulin*, together with extremely low expression levels of *CASQ2*, indicate immature Ca^2+^ handling properties in hiPSC-CMs.

The hiPSC-CMs have irregular beating rhythms resulting from immature electrophysiological properties, which hamper the precise electrophysiological analyses. The cycle length variability indices of spontaneously beating control- and CPVT-hiPSC-CMs were remarkably higher compared to those of rabbit pacemaker cells [25], indicating highly irregular contraction of hiPSC-CMs. Under spontaneously beating conditions, there were no significant differences in the frequency of diastolic Ca^2+^ waves or DADs between control- and CPVT-hiPSC-CMs. Ca^2+^ transients, ion currents (I_ca(L)_, I_to_, I_NaK_ and I_NaL_), and intracellular ion concentrations (Na^+^ and K^+^) in myocytes were reported to vary depending on pacing frequency using in-silico analyses [29, 30]. Therefore, we used electrical stimulation to perform the experiments at equal beating rates and successfully demonstrated significantly more frequent diastolic Ca^2+^ waves and DADs in CPVT-hiPSC-CMs compared to control cells. Kujala et al reported the presence of early afterdepolarizations in CPVT-hiPSC-CMs [14]; however, in our experiments, we did not identify any early afterdepolarizations during 1 or 1.5 Hz pacing. The use of 10 μM ryanodine significantly suppressed the diastolic Ca^2+^ waves of CPVT-hiPSC-CMs. This ligand fully closes ryanodine receptors at micromolar concentrations. Therefore, our results indicate that ryanodine receptors play an important role in the mechanisms of diastolic Ca^2+^ waves in CPVT-hiPSC-CMs.

Human RyR2 is a large intracellular Ca^2+^-permeable channel and consists of the N-terminal, central, and C-terminal domains [31]. The C-terminal domain is composed of the I-domain and the transmembrane domain which forms the channel pore. K201 (JTV519), a 1,4-benzothiazepine derivative, was formerly a promising candidate drug for CPVT and was thought to stabilize RyR2 by correcting defective domain-domain interactions in the N-terminal and central domains. K201 prevented the SR Ca^2+^ leak in failing cardiomyocytes by improving the defective inter-domain interaction between the N-terminal and central domains [32]. However, it failed to present cardioprotective effects on catecholamine-induced DADs and arrhythmia generation in the R4496C^+/-^ mouse model of CPVT with a mutation in the RyR2 transmembrane domain [26]. In addition, K201 was reported to be a multichannel blocker that suppressed I_Ca(L)_ and I_NCX_ (33 ± 5% reduction in the peak I_ca(L)_ with 0.3 μM K201, the concentration generally used in in vitro assays) [33]. In this context, another 1,4-benzothiazepine derivative, S107, was developed. S107 is one of the Rycals®, small molecules that prevent Ca^2+^ leakage from the SR by promoting the binding of calstabin or calstabin2 (a channel stabilizing protein) to ryanodine receptor isoform 1 or RyR2. S107 has high specificity for RyR2 and no off-target activity up to 10 μM [18]. In mouse model studies, S107 was effective for preventing ventricular arrhythmias, seizures and atrial fibrillation in CPVT [18]. In the present study, we first confirmed the efficacy of S107 in CPVT-hiPSC-CMs. Three mechanisms have been proposed to underlie leaky RyR2 channels: dissociation of a channel-stabilizing protein (calstabin2) from RyR2 [34], defective inter-domain folding of RyR2 [35], and changes in the sensitivity of RyR2 to cytosolic and luminal Ca^2+^ [36]. The augmented binding of calstabin2 to RyR2 is the only mechanism demonstrated to underlie the effect of S107. Regarding the binding site of calstabin2 on RyR2, the central domain of RyR2 has been reported to interact with calstabin2 [37]. However, Zissimopoulos and colleagues demonstrated that the C-terminal domain (containing the transmembrane domain) of RyR2 interacts with calstabin2 [38]. In our CPVT model, S107 might exert an antiarrhythmic effect by improving binding between calstabin2 and the transmembrane domain of RyR2. S107 can also prevent stress-induced cognitive dysfunction [39], the progression of heart failure [40], and muscle weakness in aging [41] or Duchenne muscular dystrophy [42]. The lead Rycal program is in phase 2 clinical studies for the treatment of heart failure and arrhythmias. These compounds may have great promise for treating patients with CPVT.

There are several limitations in this study. We analyzed CPVT-hiPSC-CMs derived from one *RyR2* mutation. It is therefore unclear whether the findings in our model would be confirmed in another CPVT hiPSC-model harboring different mutations in *RyR2* or other candidate genes. Other limitations are immature electrophysiological and structural properties of hiPSC-CMs compared to adult CMs, and a fundamental issue that we have developed a cell-based model which did not recapitulate the complex environment of a mammalian heart.

## Conclusions

We generated a stem cell-based CPVT model harboring an *RyR2* mutation in the transmembrane domain, and it successfully recapitulated the disease phenotype. Electrical pacing was highly useful for analyzing arrhythmogenic features in CPVT-hiPSC-CMs, and the antiarrhythmic effect of S107 was confirmed in this model. This CPVT hiPSC-based model assessed with electrical pacing is a powerful tool that will provide us with a better understanding of the underlying mechanisms and new approaches for screening drugs to establish personalized medicine.

## Supporting Information

S1 Table Primers used for sequencing of the human *RYR2* gene (exon 94) and qRT-PCR. (XLSX)
